# Future of Community Medicine in India

**DOI:** 10.4103/0970-0218.55301

**Published:** 2009-07

**Authors:** Gulrez Shah Azhar, Anwar Zafar Jilani

**Affiliations:** Flat B1 Room 4, Victoria Hall, 61 Eldon Street, Sheffield S1 4GX, United Kingdom; 1 Consultant in Public Health, 41 Townsgate Way, Erlam, Manchester, United Kingdom E-mail: gulrezdoc@yahoo.co.in

Sir

In spite of the impressive advances made in the field of Community Medicine in India, there is considerable confusion over its role in the future. As you have rightly pointed out in your editorial by Rajesh Kumar (Academic Community Medicine in 21^st^ Century: Challenges and Opportunities), academic growth in this subject can take either of these two directions, namely, Family Medicine or Public Health.

Moreso, this urgency to choose a direction arises because, here in the United Kingdom from where this subject had started, does not have it now. Here Family Medicine is General Practice for which in residency training, postings in conventional clinical subjects like Medicine, Surgery, Obstetrics and Gynaecology, Pediatrics, and Psychiatry is required. Moreover, the demand for Public Health is supplied with graduates from diverse backgrounds and not just medicine. This gives a richness of experience to those who are in this field. Even the postgraduate medical residency training programs and fellowship and membership examinations in the Faculty of Public Health (FPH) in the Royal Colleges are open to those who do not have medicine as a subject in graduation.

The basic shortcoming of public health in India, which we feel, is that those with training in public health are not in the position to make decisions. As conventionally in the government setup these crucial positions are handled by bureaucrats and ministers who do not have the training or experience in public health.

It is surprising to us that in these times of rapid growth, when we are continually made to believe that we are becoming superpowers, the health situation in our country is so dismal. That no effort is being made to correct this state of affairs is evident in the amount of funding which the government provides for our health. Only cosmetic and piecemeal solutions are suggested and no attempt is being made to correct systemic faults. It is so strange that Indian doctors coming here are working and training in *National Health Service* (NHS), while in India, after doing MBBS, they have to sit at home for years and prepare for postgraduate entrance examinations; this is a major waste of manpower for us. The developed countries gain at our expense, no one likes to leave their home and country by choice, including doctors. The salaries paid to resident doctors in some states are laughable (the defense being that MD is a course; while universally residency is considered to be a training post) and even the democratic right to protest is taken away by laws such as Essential Services Maintenance Act. Instead of improving the jobs, salaries, and working conditions, what is done is ad-hoc appointments, and suggestions of inclusion of semi skilled/unskilled practitioners in government programs. Instead of improving the total healthcare infrastructure, vertical programs are launched for one condition at a time.

The current postgraduates in community medicine are at a loss to understand their role in mitigating the health situation in the country. The future to them appears to be only in academics, as responsible public health positions in international organizations are open to everyone and not just doctors of community medicine. Furthermore, community medicine standing at crossroads between family medicine and public health since a long time is not too helpful either.

We therefore suggest that the demand for family medicine specialists be supplied by a massive increase in the number of residency positions for MBBS graduates in all institutions on the pattern of general practice training in the UK. Community medicine should be integrated to public health and include the elements of statistics, economics, demography, informatics, epidemiology and disease prevention, gender and MCH, occupational and environmental health, research methods (primary and secondary including systematic reviews), and management, as further specializations. This should be open to both medical and nonmedical graduates.

Most importantly, the government should realise that mere cosmetic changes in the health system will only do more harm than good in the long run. It needs to massively increase the funding in healthcare, overhaul the existing healthcare system in line with the future demands and recognize and utilize the knowledge and skills of public health professionals at every stage in the decision making process.

